# Tetramethylpyrazine Analogue T-006 Protects Neuronal and Endothelial Cells Against Oxidative Stress via PI3K/AKT/mTOR and Nrf2 Signaling

**DOI:** 10.3390/antiox13101272

**Published:** 2024-10-21

**Authors:** Guiliang Zhang, Zirong Liang, Yuqiang Wang, Zaijun Zhang, Pui-Man Hoi

**Affiliations:** 1 State Key Laboratory of Quality Research in Chinese Medicine, Institute of Chinese Medical Sciences, University of Macau, Macao SAR, China; yc17527@um.edu.mo (G.Z.); yc37571@um.edu.mo (Z.L.); 2 Department of Pharmaceutical Sciences, Faculty of Health Sciences, University of Macau, Macao SAR, China; 3 Institute of New Drug Research, International Cooperative Laboratory of Traditional Chinese Medicine Modernization and Innovative Drug Development of Chinese Ministry of Education, Jinan University College of Pharmacy, Guangzhou 510632, China; yqwang@jnu.edu.mo (Y.W.); zaijunzhang@jnu.edu.cn (Z.Z.); 4 Guangdong-Hong Kong-Macau Joint Laboratory for Pharmacodynamic Constituents of TCM and New Drugs Research, and Guangdong Province Key Laboratory of Pharmacodynamic Constituents of TCM and New Drugs Research, Jinan University College of Pharmacy, Guangzhou 510632, China; 5 International Cooperative Laboratory of Traditional Chinese Medicine Modernization and Innovative Drug Development of Chinese Ministry of Education (MOE), Jinan University College of Pharmacy, Guangzhou 510632, China

**Keywords:** tetramethylpyrazine analogue T-006, oxidative cytotoxicity, excessive glutamate, OGD/R, PI3K/AKT, Nrf2/HO-1, mTOR

## Abstract

Background: T-006, a novel neuroprotective derivative of tetramethylpyrazine (TMP), exhibits multifunctional neuroprotective properties. T-006 has been shown to improve neurological and behavioral functions in animal models of ischemic stroke and neurodegenerative diseases. The present study aims to further elucidate the mechanisms underlying the protective effects of T-006 against oxidative injuries induced by glutamate or hypoxia. Methods: Mouse hippocampal HT22 cells were used to evaluate the neuroprotective effects of T-006 against glutamate-induced injuries, while mouse brain endothelial bEnd.3 cells were used to evaluate the cerebrovascular protective effects of T-006 against oxygen-glucose deprivation followed by reperfusion (OGD/R)-induced injuries. The 3-(4,5-dimethylthiazol-2-yl)-2,5-diphenyltetrazolium bromide (MTT) assay and flow cytometry were used to measure cell viability and oxidative stress. Western blot and immunofluorescence analyses of protein expression were used to study cell signaling pathways. Results: T-006 exhibited significant protective effects in both oxidative injury models. In HT22 cells, T-006 reduced cell death and enhanced antioxidant capacity by upregulating mTOR and nuclear factor erythroid 2-related factor 2/Heme oxygenase-1 (Nrf2/HO-1) signaling. Similarly, in bEnd.3 cells, T-006 reduced oxidative injuries and preserved tight junction integrity through Nrf2/HO-1 upregulation. These effects were inhibited by LY294002, a Phosphoinositide 3-kinase (PI3K) inhibitor. Conclusions: T-006 may exert its neuroprotective and cerebrovascular protective effects via the regulation of PI3K/AKT-mediated pathways, which facilitate downstream mTOR and Nrf2 signaling, leading to improved cell survival and antioxidant defenses.

## 1. Introduction

Ischemic stroke is characterized by the acute interruption of blood flow to the central nervous system (CNS), typically caused by blockage or narrowing of arteries. The depletion of oxygen and glucose disrupts ATP synthesis and causes energy deficiency, resulting in impaired ion homeostasis and acid–base imbalance [1]. These dysfunctions rapidly lead to a series of deleterious cellular events, including excitotoxicity, mitochondrial dysfunction, oxidative stress, cell death processes, and inflammatory responses [1]. The acute inflammatory response following ischemic stroke triggers the activation and migration of immune cells such as microglia and peripheral leukocytes to the site of injury and releases proinflammatory cytokines and chemokines. In addition, ischemic conditions lead to reactive oxidative species (ROS) and free radical generation, which not only further stimulate inflammatory pathways and exacerbate tissue damage, but also induce cellular injuries to neurons, glia, and endothelial cells in the brain [2,3]. In neurons, oxygen deprivation leads to ionic imbalance and membrane depolarization, triggering pathological glutamate release to the extracellular space [2]. Glutamate, being a major neurotransmitter in the brain, activates both ionotropic (iGluRs) and metabotropic (mGluRs) receptors for various physiological functions such as synaptic transmission, neuronal excitability, and plasticity [4,5]. However, excessive glutamate causes toxicity in neuronal cells in two ways, with the overactivation of iGluRs causing excitotoxicity [6] and the depletion of the antioxidant glutathione, causing oxidative injury [7]. Extracellular glutamate accumulation reverses the action of cystine/glutamate antiporter in neurons, ultimately decreasing the intracellular glutathione level and antioxidant defense capacity, causing serious oxidative injury [8]. Glutamate toxicity has been strongly implicated in the pathophysiology of numerous neurodegenerative diseases including amyotrophic lateral sclerosis (ALS), Alzheimer’s disease (AD), and Parkinson’s disease (PD) [9,10,11].

Apart from neuronal cells, the brain endothelial cells that line the blood vessels of the CNS are also vulnerable and the first to respond to oxidative insults in cerebral ischemia [12]. Brain endothelial cells, as key components of the blood-brain barrier (BBB), have highly specialized physiological features such as the presence of tight junctions (TJs) to maintain barrier integrity for regulating the passage of substances between blood and brain [13]. Damage to endothelial cells induced by ischemia can result in the disruption of TJs, leading to increased BBB permeability and the onset of brain edema, triggering inflammatory responses and exacerbating tissue damage [14]. Therefore, protecting endothelial cells and maintaining BBB integrity are pivotal strategies for mitigating ischemic stroke [15,16].

Phosphoinositide 3-kinase/Protein kinase B (PI3K/AKT) signaling pathway is a crucial pathway for cell survival, growth, metabolism, apoptosis, and other physiological activities [17]. The dysregulation of PI3K/AKT has been strongly associated with pathophysiological processes in ischemic stroke and glutamate toxicity [17]. AKT regulates cell growth through mTOR signaling [18] and is critically involved in the regulation of Nrf2, a transcription factor central to cellular oxidative defense [19]. Mounting evidence suggests that a deficiency in Nrf2 signaling suppresses the induction of antioxidant genes and increases susceptibility to oxidative damage [20]. Furthermore, Nrf2 activation has been shown to protect BBB integrity by maintaining endothelial physiological and barrier functions [21].

Previous studies have demonstrated that T-006, a derivative of tetramethylpyrazine (TMP), significantly attenuated neuronal cell death induced by excessive glutamate and oxidative stress in rat cortical neurons and cerebellar granule neurons (CGN) [22,23]. Studies in AD and PD animal models showed that T-006 provided extensive neuroprotection and therapeutic effects [24,25,26,27]. In particular, T-006 promoted neuronal regeneration and restored neurological functions in a rat model of stroke [28]. Therefore, in the present study, we aim to further investigate the antioxidant potential of T-006 as a therapeutic candidate against oxidative injuries in neuronal and endothelial cells in ischemic stroke condition, and to dissect the underlying molecular mechanisms.

## 2. Materials and Methods

### 2.1. Chemicals and Reagents

T-006 was designed and synthesized by the Jinan University (Guangzhou, China). Glutamate and tetramethylpyrazine were purchased from Sigma-Aldrich (St. Louis, MO, USA). 3-(4,5-dimethylthiazol-2-yl)-2,5-diphenyltetrazolium bromide (MTT), PI3K inhibitor LY294002 and 4′,6-diamidino-2-phenylindole (DAPI) were purchased from Beyotime (Shanghai, China). Dulbecco’s modified Eagle’s medium (DMEM), fetal bovine serum (FBS), and penicillin/streptomycin (PS) were obtained from Gibco (Waltham, MA, USA). The Annexin V-FITC/propidium iodide (PI) apoptosis detection kit was obtained from Mei5bio (Beijing, China). 5-(and-6-)-chloromethyl-2′,7′-dichlorodihydrofluorescein diacetate and acetyl ester (CM-H2DCFDA), BCA kit and goat anti-rabbit IgG with Alex Fluor 488 were purchased from Invitrogen (Carlsbad, CA, USA). Chloroquine was purchased from MedChemExpress (Monmouth Junction, NJ, USA). Primary antibodies and horseradish peroxidase (HRP)-conjugated secondary antibodies were purchased from Cell Signaling Technology (Danvers, MA, USA) and Proteintech (Wuhan, China). 

### 2.2. Cell Cultures

Mouse hippocampal neuronal cell line HT22 was generously provided by Prof. Yonghua Zhao (Institute of Chinese Medical Sciences, University of Macau). Mouse brain endothelial cell line bEnd.3 cells were purchased from the American Type Culture Collection (ATCC). Cells were cultured in high glucose DMEM medium supplemented with 10% FBS and 1% PS and maintained in a humidified incubator at 37 °C with 5% CO_2_.

### 2.3. Glutamate or OGD/R-Induced Oxidative Stress Models

For the neuronal cell model, HT22 cells were challenged by high concentration of glutamate (10 mM) for 24 h to induce oxidative cytotoxicity. T-006 (1, 3, and 10 μM) or TMP (10 μM) were administered as cotreatment in HT22 cells. For brain endothelial cell model, bEnd.3 cells were challenged by OGD/R conditions as previously reported [29]. Briefly, bEnd.3 cells were washed with PBS and subjected to glucose-free DMEM in anoxia (95% N_2_ and 5% CO_2_) at 37 °C for 4 h to mimic the glucose deprivation and hypoxic state in ischemia, followed by normoxia (95% air and 5% CO_2_) for 12 h to mimic the reperfusion process. The control group was maintained in an aerobic normoxia environment (95% air and 5% CO_2_) with high-glucose DMEM. T-006 or TMP were administered as pretreatment (6 h) in bEnd.3 cells and were present during OGD/R. In experiments where PI3K inhibitor LY294002 was used, the inhibitor was administered 2 h before oxidative challenge (glutamate or OGD/R). In experiments where chloroquine (10 μM) was used, it was administered with glutamate for 24 h.

### 2.4. Cell Viability Assay

HT22 cells were seeded in 96-well plates at a density of 8 × 10^3^ cells/well. bEnd.3 cells were seeded overnight in a 96-well plate at a density of 1 × 10^4^ cells/well. MTT solution (0.5 mg/mL) was added to each well and incubated for 4 h following the removal of the culture medium. Formazan crystals were dissolved in each well by adding 100 µL of DMSO. The absorbance of each well at 570 nm was measured using a microplate reader.

### 2.5. Quantitative Analysis of Apoptotic Cells by Flow Cytometry

FITC-Annexin V apoptosis detection kit was used to quantify apoptosis according to the manufacturer’s instructions by flow cytometry. The cells were seeded at a density of 1 × 10^5^ cells/well in 12-well plates and harvested after drug treatment in the glutamate-induced model. Cells were washed and stained with FITC-annexin V and PI for 15 min at room temperature in the darkness, followed by analysis using flow cytometry.

### 2.6. Reactive Oxygen Species (ROS) Detection

After drug treatment and experiments, CM-H2DCFDA (10 μM) was added to each well, and the cells were incubated at 37 °C for 30 min in darkness, followed by washing with PBS twice. Fluorescence intensity was measured by flow cytometry.

### 2.7. Measurement of TEER in Endothelial Monolayer

bEnd.3 cells were cultured in transwell inserts with culture medium, 300 μL in the upper compartment and 1 mL in the lower compartment. Transendothelial electrical resistance (TEER) of endothelial monolayer was measured using electrodes and calculated by using the following equation:

TEER (Ω.cm^2^) = (Resistance (Ω) − Background Resistance (Ω)) × Membrane area (cm^2^)

### 2.8. Immunofluorescence Staining of Tight Junction Protein

bEnd.3 cells were fixed by 4% paraformaldehyde at room temperature for 20 min, permeabilized by adding 0.2% Triton X-100 for 15 min, and blocked with the 5% BSA for 1 h. The cells were then incubated with primary antibodies against ZO-1 (Proteintech, Wuhan, China, 21773-1-AP; 1:500) at 4 °C overnight. After washing with PBS 3 times, cells were incubated with secondary antibodies (1:1000) labeled with Alexa Fluor-488 for 2 h at room temperature. Nuclei were counterstained with DAPI for 10 min. Cell sections were imaged by fluorescence microscopy (Leica, Wetzlar, Germany).

### 2.9. Western Blotting

Total protein was extracted using RIPA lysis buffer (Beyotime, Shanghai, China) containing protease and phosphatase inhibitor cocktail. Protein concentrations were measured by BCA kit (Thermo Scientific, Waltham, MA, USA). Equal amounts of proteins (30–40 µg) were separated by 10% SDS-PAGE gels and transferred to 0.22 µM polyvinylidene difluoride (PVDF) membranes (Millipore, Burlington, MA, USA). After blocking with 5% non-fat milk for 90 min at room temperature, membranes were incubated with the appropriate primary antibody overnight at 4 °C, including ZO-1 (Proteintech, Wuhan, China, 21773-1-AP), Nrf2 (Proteintech, Wuhan, China, 16396-1-AP), HO-1 (Proteintech, Wuhan, China, 10701-1-AP), p-AKT (CST, Danvers, MA, USA, 4060S), AKT (CST, Danvers, MA, USA, 9272S), p-mTOR (CST, Danvers, MA, USA, 2971S), mTOR (CST, Danvers, MA, USA, 5536S), LC3 I/II (CST, Danvers, MA, USA, 4108S), SQSTM1/p62 (CST, Danvers, MA, USA, 23214S), and β-actin (Proteintech, Wuhan, China, 20536-1-AP). After washing 3 times with PBS, membranes were incubated with HRP-conjugated secondary antibodies at room temperature for 2 h. After repeated washes with TBST, proteins were visualized using an ECL advanced Western blotting detection kit (Thermo Scientific, Waltham, MA, USA). Photos of the protein bands were taken using Image Lab Version 5.1 (Bio-Rad, Hercules, CA, USA) and densitometry measurements of band intensity in the Western blots were performed using Bio-Rad Image 5.1 software.

### 2.10. Statistical Analysis

Statistical analysis was performed using GraphPad Prism software version number 8.0. Results are presented as mean ± SEM from 3 to 4 independent experiments (n = 3–4), each run at least in triplicate. All data followed normal distribution. Data were analyzed using one-way ANOVA followed by Tukey’s post-hoc test. Statistical significance was considered at *p* < 0.05. 

## 3. Results

### 3.1. T-006 Protected HT22 Cells Against Glutamate-Induced Oxidative Cytotoxicity

**Figure 1:**
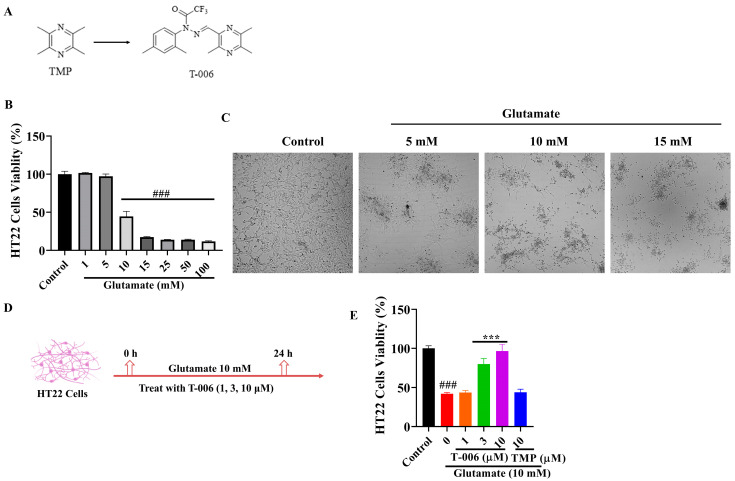
Effects of T-006 in HT22 cells challenged by excessive glutamate. (**A**) Chemical structure of TMP and T-006. (**B**) Cell viability of HT22 cells treated with different concentrations of glutamate as measured by MTT assay. (**C**) Representative images of HT22 cells morphology under glutamate challenge (scale bar, 50 μm). (**D**) Schematic diagram of experiment protocol. HT22 cells were incubated with T-006 (1, 3, and 10 μM) or TMP (10 μM) for 24 h in the presence of glutamate. (**E**) Cell viability of HT22 cells treated with different concentrations of T-006 and TMP in the presence of glutamate (10 mM) as measured by MTT assay. Data are shown as mean ± SEM from at least three independent experiments (n > 3) and analyzed by one-way ANOVA followed by Tukey test. ^###^ *p* < 0.001 vs. control group, *** *p* < 0.001 vs. glutamate-induced group.

Previous studies showed that T-006, which is a derivative of tetramethylpyrazine (TMP) (Figure 1A), significantly attenuated cell death induced by excessive glutamate and oxidative stress in rat cortical neurons and cerebellar granule neurons (CGN) [22,23]. In ischemic stroke, oxygen and glucose deprivation leads to energy depletion and ionic imbalance, causing cell membrane depolarization and calcium overload in neuronal cells, triggering aberrant glutamate release and extracellular glutamate accumulation [30]. In the present study, we further explored the underlying mechanisms of T-006 against glutamate-induced oxidative cytotoxicity in the mouse hippocampal neuronal cell line HT22. Based on previous studies in various cultured neuronal cells such as CGN and PC12 [22,23,24,25,26], the concentrations of T-006 (1, 3, 10 μM) were demonstrated as safe and pharmacologically effective and were selected for further investigation in the present study. MTT assay was used to monitor the viability of HT22 cells challenged with various concentrations of glutamate. It was observed that treatment with 10 mM glutamate for 24 h reduced the cell viability of HT22 cells by between 40–70% when compared to the control (Figure 1B) and induced changes in the cellular morphology, including nuclear condensation and cellular shrinkage, as observed by microscopy (Figure 1C). Cotreatment with T-006 (1, 3, and 10 μM) dose-dependently protected HT22 cells exposed to glutamate, whereas the effect of TMP was negligible (Figure 1E).

### 3.2. T-006 Attenuated Glutamate-Induced ROS Production and Apoptosis in HT22 Cells

**Figure 2:**
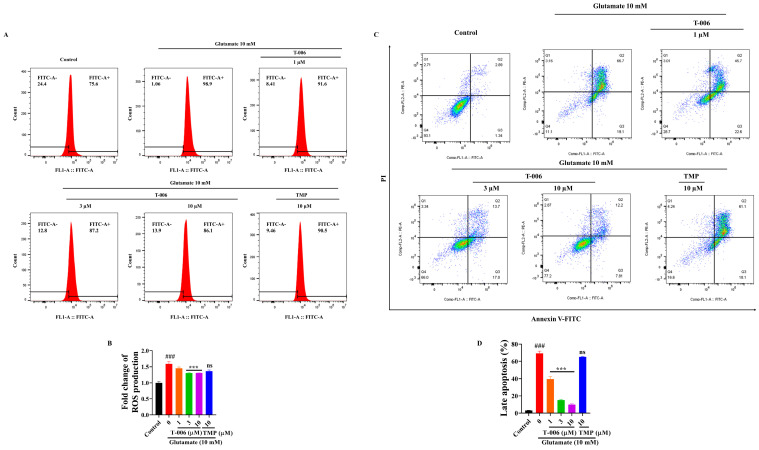
T-006 inhibited glutamate-induced ROS production and apoptosis in HT22 cells. (**A**,**B**) Quantitative analysis of ROS generation by flow cytometry with H2DCFHDA. (**C**,**D**) Quantitative analysis of apoptosis by flow cytometry with Annexin V-FITC/PI. Data are shown as mean ± SEM from at least three independent experiments (n > 3) and analyzed by one-way ANOVA followed by Tukey test. ^###^ *p* < 0.001 vs. control group, *** *p* < 0.001 vs. glutamate-induced group. “ns” stands for “not significant”.

The effects of T-006 against glutamate-induced apoptosis and intracellular ROS production in HT22 cells were evaluated by flow cytometry. The results showed that glutamate exposure induced a significant increase in ROS production in HT22 cells, and T-006 cotreatment was able to inhibit ROS generation (Figure 2A,B). Similarly, as shown in Figure 2C,D, exposure to glutamate increased the percentage of late apoptotic cells to about 70% compared to the control. In contrast, T-006 cotreatment dose-dependently attenuated the apoptotic population, while TMP did not have any effect. HT22 cells are sensitive to high concentrations of extracellular glutamate despite that fact that these cells lack ionotropic glutamate receptors (iGluRs) and previous studies showed that glutamate evokes oxidative injuries in HT22 cells involving necrotic and apoptotic processes [31,32]. Excessive extracellular glutamate inhibits cysteine uptake by reversing the action of the cysteine/glutamate antiporter and leads to the depletion of glutathione levels, resulting in cell vulnerability to ROS, calcium dysregulation, and lipid peroxidation [7]. ROS-induced damage is further exacerbated by Bid-dependent mitochondrial dysfunction, leading to mitochondrial fragmentation and the release of apoptosis-inducing factor (AIF) into the nucleus, resulting in caspase-independent apoptosis [33]. Taken together, T-006 cotreatment activated cellular antioxidant mechanisms by upregulating the Nrf2/HO-1 pathway, enhancing cellular ability to scavenge ROS and protect against oxidative damage.

### 3.3. T-006 Enhanced mTOR Signaling and Suppressed Glutamate-Induced Autophagic Cell Death in HT22 Cells

**Figure 3:**
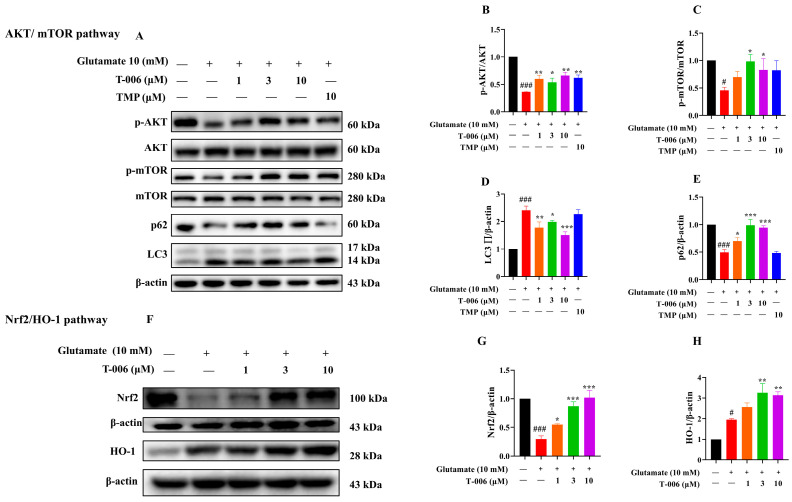
T-006 induced protective effect via AKT-mediated mTOR and HO-1 signaling in glutamate-challenged HT22 cells. (**A**) Representative immunoblots of AKT, mTOR, LC3, and p62 proteins. (**B**–**E**) Quantitative analysis for p-mTOR, p-AKT, LC3-I/II, and p62 proteins levels (n = 3–4). (**F**) Representative immunoblots of Nrf2 and HO-1 proteins. (**G**,**H**) Quantitative analysis for Nrf2 and HO-1 (n = 3–4). Data are shown as mean ± SEM and analyzed by one-way ANOVA followed by Tukey test. ^#^ *p* < 0.05 and ^###^ *p* < 0.001 vs. control group; * *p* < 0.05, ** *p* < 0.01 and *** *p* < 0.001 vs. glutamate-induced group.

A previous study showed that glutamate-induced oxidative toxicity involved autophagic cell death in HT22 cells [34]. Thus, we wanted to investigate if T-006 could rescue cell death by inhibiting autophagy. We assessed the induction of autophagy in glutamate-treated HT22 cells by measuring the conversion of microtubule-associated protein 1 light chain 3 (LC3) from the cytoplasmic form LC3-I (17 kDa) to the autophagosome-bound form LC3-II (14 kDa), and the expression of LC3 binding protein p62 (also known as SQSTM1) [35]. In addition, we evaluated the expression and activation of the mammalian target of rapamycin (mTOR), which is involved in the suppression of autophagy via the PI3K/AKT/mTOR pathway [36]. As shown in Figure 3A, glutamate treatment (10 mM for 24 h) significantly enhanced autophagy, as indicated by decreased p-mTOR, increased conversion of LC3-I to LC3-II, and decreased p62 levels (Figure 3C–E). Western blotting also showed a significant reduction in p-AKT levels in the glutamate-exposed group compared to the control (Figure 3B). Cotreatment of T-006 significantly increased mTOR activation, attenuated LC3-I/LC3-II conversion, and caused p62 reduction. These data suggested that T-006 significantly inhibited autophagic cell death induced by glutamate.

### 3.4. T-006 Protected HT22 Cells Against Glutamate-Induced Oxidative Toxicity via PI3K/AKT-Mediated mTOR and Nrf2/HO-1 Signaling Pathways

**Figure 4:**
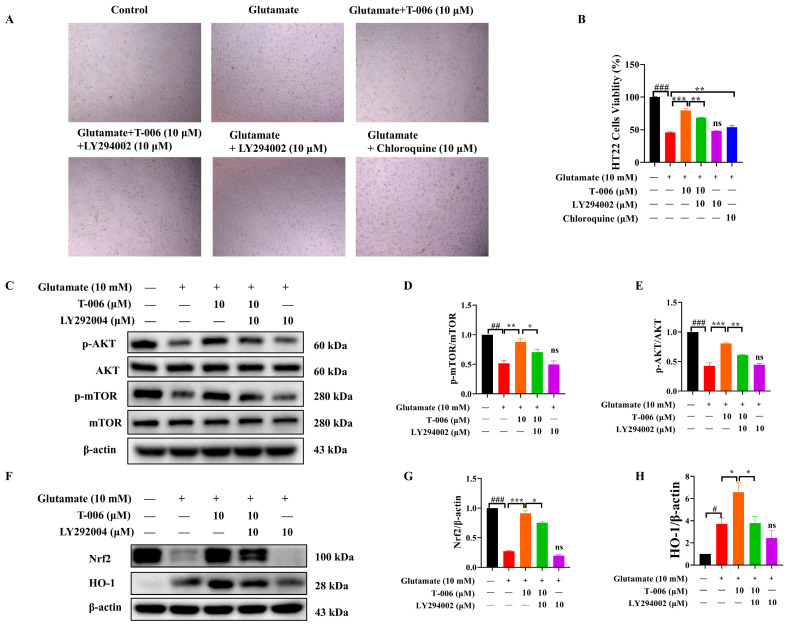
T-006 induced protective effects in glutamate-challenged HT22 cells was inhibited by LY294002. (**A**) Representative images of cell morphology with bright field microscope (scale bar 100 μm). (**B**) Cell viability was detected by MTT assay (n = 6). (**C**) Representative immunoblots of AKT and mTOR proteins. (**D**,**E**) Quantitative analysis for p-mTOR and p-AKT (n = 3–4). (**F**) Representative immunoblots of Nrf2 and HO-1 proteins. (**G**,**H**) Quantitative analysis for Nrf2 and HO-1 (n = 3–4). Data are shown as mean ± SEM and analyzed by one-way ANOVA followed by Tukey test. ^#^ *p* < 0.05, ^##^ *p* < 0.01, and ^###^ *p* < 0.001 vs. control group; * *p* < 0.05, ** *p* < 0.01 and *** *p* < 0.001 vs. glutamate-induced group. “ns” stands for “not significant”.

Activation of the PI3K/AKT signaling pathway can serve as a protective mechanism to inhibit autophagy and alleviate oxidative stress and the apoptosis of neuronal cells in ischemic stroke [37]. Therefore, we assessed the effects of T-006 on the activation of related pathways. Our results showed that the PI3K inhibitor LY292004 inhibited the protective effects of T-006 (Figure 4A,B). Chloroquine, an autophagy inhibitor [38], also inhibited glutamate-induced cell death, but its effect was inferior to that of T-006 (Figure 4A,B). Exposure to glutamate downregulated the levels of p-AKT and p-mTOR in HT22 cells, which were restored by T-006 in a LY292004-sensitive manner (Figure 4D,E). Next, we assessed Nrf2/HO-1, a key cellular antioxidant regulatory pathway downstream of PI3K/AKT [39]. We observed that cotreatment of T-006 dose-dependently increased the protein levels of Nrf2 and HO-1 (Figure 3F), which was inhibited by LY292004 (Figure 4F). Interestingly, we observed that glutamate exposure (10 mM for 24 h) in HT22 cells resulted in a downregulated level of Nrf2 and an upregulated level of HO-1 compared to the control (Figure 3F). Published studies have generally reported a moderate decrease in Nrf2 and HO-1 levels in HT22 cells after glutamate challenge [40,41], so this upregulation of HO-1 was unexpected. However, previous studies usually used lower glutamate concentrations or shorter incubation periods (e.g., 5 mM for 12 h). We proposed this elevation reflected a stress-response mechanism activated by glutamate, leading to upregulation of HO-1 as a protective measure against oxidative stress and potential cellular damage. Taken together, these data suggested that T-006 mediated anti-autophagic and anti-oxidative effects to protect neuronal cells against glutamate-induced oxidative cytotoxicity, at least in part via the PI3K/AKT-mediated mTOR and Nrf2/HO-1 signaling pathways.

### 3.5. T-006 Protected bEnd.3 Cells Against OGD/R-Induced Oxidative Cytotoxicity

**Figure 5:**
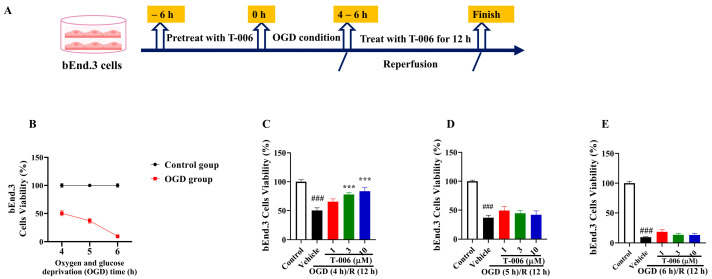
Effects of T-006 in bEnd.3 challenged by OGD/R. (**A**) Schematic diagram experimental protocol. (**B**) The effect of different OGD (oxygen and glucose deprivation) times on cell viability. (**C**–**E**) Cell viability was detected by an MTT assay. Data are shown as mean ± SEM (n > 3) and analyzed by one-way ANOVA followed by Tukey test. ^###^ *p* < 0.001 vs. control group, *** *p* < 0.001 vs. vehicle group.

Next, we further evaluated the protective effects of T-006 against oxidative injury in brain endothelial cells, which are key components of the BBB. Oxygen and glucose deprivation in ischemic stroke triggers the generation of oxidative stress, causing disruptions in endothelial functions and BBB integrity [42], affecting the surrounding cellular environment, and oxidative damage is further exacerbated after reperfusion. We employed the mouse brain endothelial cell line bEnd.3 exposed to oxygen glucose deprivation for various time periods (4 to 6 h) followed by reoxygenation (12 h) (OGD/R) as a cellular model to simulate oxidative injuries (Figure 5A). As shown in Figure 5B, OGD/R (4 h OGD followed by 12 h) reduced the endothelial cell viability by ~50%, while longer OGD periods (5 h and 6 h) further reduced cell viabilities by ~75% and ~95%. The pretreatment of T-006 (6 h) dose-dependently ameliorated endothelial cell death against OGD/R (4 h OGD followed by 12 h) (Figure 5C). However, T-006 was unable to protect endothelial cells when OGD periods were longer (Figure 5D,E).

### 3.6. T-006 Attenuated Brain Endothelial Cell Dysfunction via AKT/Nrf2/HO-1 Pathway

**Figure 6:**
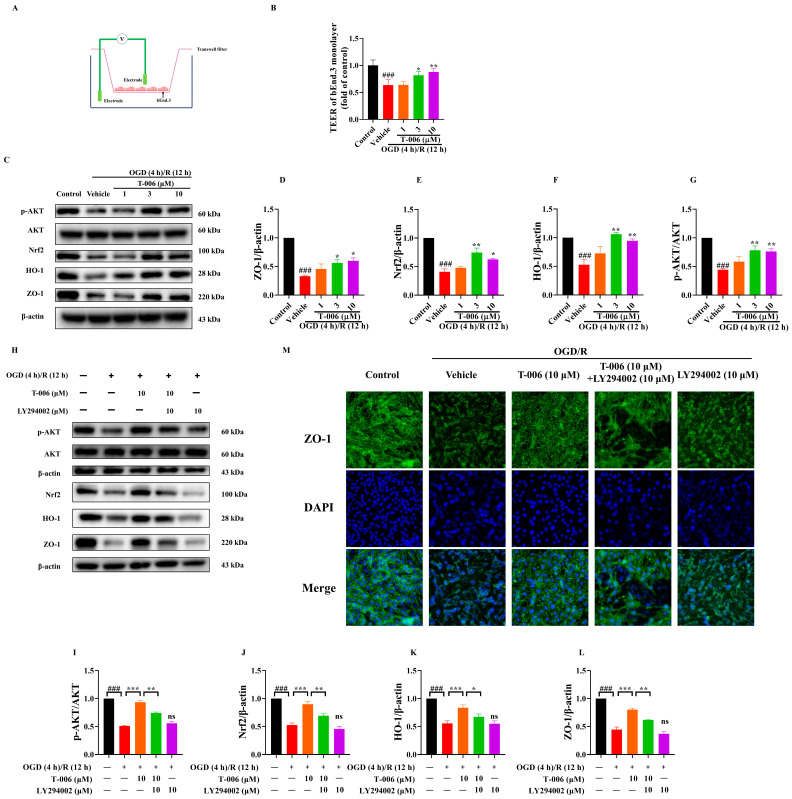
T-006 protected bEnd.3 cells against oxidative injuries and tight junction disruption induced by OGD/R. (**A**,**B**) Measurement of TEER of endothelial monolayer. (**C**,**H**) Representative immunoblots of AKT, Nrf2, HO-1, and ZO-1. (**D**–**G**,**I**–**L**) Quantitative analysis of Western blot results (n = 3–4). (**M**) Representative immunofluorescence image (scale bar 25 μm) of staining of tight junction protein (ZO-1). Data are shown as mean ± SEM and analyzed by one-way ANOVA followed by Tukey test. ^###^ *p* < 0.001 vs. control group; * *p* < 0.05, ** *p* < 0.01 and *** *p* < 0.001 vs. vehicle group. “ns” stands for “not significant”.

The Nrf2/HO-1 signaling is one of the most critical antioxidant and self-defense mechanisms and is of particular importance in endothelial homeostasis [43]. Therefore, we investigated if the protection of bEnd.3 against OGD/R was mediated by Nrf2/HO-1 pathway. We investigated if the enhanced transcriptions of Nrf2 and HO-1 were mediated via PI3K/AKT pathway. In the presence of LY294002 (PI3K inhibitor), T-006 induced upregulations of p-AKT, Nrf2 and HO-1 were markedly attenuated, as shown by Western blotting (Figure 6H,J,K). In addition, we examined if T-006 also enhanced endothelial barrier function. OGD/R significantly decreased the barrier tightness of the bEnd.3 monolayer, as evaluated by transendothelial electrical resistance (TEER). Our results showed that T-006 pretreatment prevented the OGD/R-mediated loss of barrier integrity in a dose-dependent manner (Figure 6B). The expression of endothelial tight junction proteins ZO-1 was reduced markedly by OGD/R in bEnd.3, as shown by Western blotting and immunofluorescent staining (Figure 6H,M). T-006 pretreatment significantly prevented the loss of ZO-1 in a dose-dependent manner (Figure 6C,D). Meanwhile, LY294002 inhibited the enhancement of ZO-1 expression mediated by T-006 (Figure 6L). Taken together, these data suggested that T-006 protected brain endothelial cells against oxidative damages and integrity loss by upregulating HO-1 and ZO-1.

## 4. Discussion

Tetramethylpyrazine (TMP), an active ingredient in the Chinese herb *Ligusticum wallichii* Franch (Chuanxiong), has been widely used for the prevention and treatment of ischemic cardiovascular and cerebrovascular diseases, and the benefits of TMP are largely attributed to its antioxidative properties [44]. However, the therapeutic potential of TMP is limited by its low bioavailability and rapid metabolism [45]. T-006 is a derivative of TMP with improved drug absorption, and previous studies by our team demonstrated that T-006 displayed multifunctional neuroprotective effects in AD and PD mouse models [20,21,22,23,24,25,26,27]. T-006 has also been shown to significantly improve cognitive functions and promote neurogenesis in a rat model of ischemic stroke [28]. In addition, T-006 demonstrates better bioavailability and a longer half-life compared to its parent compound TMP, allowing a better dosing strategy, improved therapeutic efficacy, and reduced toxicity or side effects due to excessive medication concentrations.

In the present study, we employed two cell models, glutamate-challenged HT22 cells and OGD/R-challenged bEnd.3 cells, to evaluate the neuroprotective and cerebrovascular protective effects of T-006 against oxidative injuries. In ischemic stroke, the abnormal release and accumulation of extracellular glutamate is a major cytotoxic event causing neuronal cell death [30], and glutamate-challenged HT22 cells are a well-established cellular model for screening neuroprotective compounds against oxidative stress [46]. In addition, oxidative stress is a common pathological feature in various neurodegenerative diseases [47]. Excessive glutamate causes toxicity in neuronal cells in two ways: (1) excitotoxicity due to the overactivation of ionotropic glutamate receptors (iGluRs) such as NMDA receptor [6], and (2) oxidative injury due to the depletion of antioxidant glutathione [7]. It is reported that the expression of iGluRs is low in undifferentiated HT22 cells [48]. Glutamate toxicity in HT22 cells is primarily triggered by non-receptor mediated oxidative injury associated with disrupted activity of the cystine/glutamate antiporter, leading to impaired cystine uptake and the subsequent cellular depletion of glutathione [7]. The resulting ROS, lipid peroxidation, and mitochondrial dysfunction all contribute to oxidative neuronal injuries and cell death. Our results showed that T-006 significantly protected HT22 cells against ROS production, apoptosis, and necrosis in a dose-dependent manner (1 to 10 μM), whereas TMP (10 μM) produced a negligible effect. Further investigation of the underlying mechanisms showed that T-006 protected HT22 cells against cell death via mTOR and Nrf2 signaling, indicating T-006 regulated autophagy and an antioxidative stress response.

It is noteworthy that autophagy plays a pivotal role in neuronal cellular homeostasis regulating protein quality control and damaged organelles clearance [49]. It is reported that prolonged glutamate accumulation causes the overactivation of autophagy [34]. Excessive autophagy can lead to detrimental outcomes such as essential protein degradation, organelle dysfunction, energy depletion, impaired cellular signaling, and inflammatory responses [50]. One of the key regulators of autophagy is mTOR, which also regulates various cellular processes including metabolism, proliferation, survival, and immune responses to maintain cellular homeostasis. Under normal nutrient-rich conditions, mTOR is activated to promote anabolic processes such as protein synthesis and inhibit degradative processes such as autophagy, and the dysregulation of mTOR signaling has been observed in neurodegenerative diseases, making autophagy modulators attractive therapeutic agents for disease intervention. For example, rapamycin and chloroquine are two well-known modulators of the autophagy pathway with different working mechanisms. Rapamycin, a potent autophagy inducer, inhibits mTOR signaling and leads to the promotion of enhanced cellular degradation of damaged organelles and proteins [36]. In contrast, chloroquine acts as an autophagy inhibitor by preventing the fusion of autophagosomes with lysosomes, thereby blocking autophagic flux and the degradation process [51]. These differential modulation mechanisms highlight the complexity of autophagy regulations and its implications for therapeutic strategies. Indeed, our data showed that T-006 produced its effects through both mechanisms. T-006 significantly promoted mTOR signaling, as indicated by the reduction in LC3-I/LC3-II conversion, and decreased autophagic flux, as indicated by the increased level of p62 (also known as SQSTM1), indicating attenuated autophagic cell death in HT22 challenged by glutamate. When compared to its parent compound TMP, T-006 exhibited more potent protective effects. T-006 (10 μM) protected > 90% of HT22 cells from glutamate-induced oxidative cytotoxicity and apoptosis, while TMP (10 μM) did not exhibit protection (Figure 1E and Figure 2D). T-006 was also much more effective at activating mTOR signaling than TMP (Figure 3D,E). Meanwhile, T-006 and TMP were similar in their ability to activate Nrf2/HO-1 signaling (Figure 3C,H). Thus, our data indicate that while TMP can promote a certain level of antioxidant response, T-006 is capable of enhancing both antioxidant and anti-apoptotic responses.

The interplay between mTOR and Nrf2 plays a significant role in neuroprotection, particularly in the context of oxidative stress damages. One of the most evident connections between mTOR and Nrf2 is the antioxidant response element (ARE) sequence in the mTOR promoter region linking Nrf2. This allows Nrf2 to directly regulate mTOR genetic expression in a PI3K/AKT-dependent manner [52]. This highlights the intricate system of the Nrf2/ARE signaling pathway in mTOR function. Furthermore, the activation of mTOR can induce Nrf2 expression. Under normal physiological conditions, the transcription of Nrf2 is restricted by Keap1, which acts as a negative regulator by sequestering Nrf2 in the cytoplasm. The activation of mTOR can induce the phosphorylation of p62 and promote p62-mediated Keap1 degradation, allowing Nrf2 translocation into nucleus. Hence, mTOR signaling enhances cellular antioxidant responses through the induction of Keap1-Nrf2 pathway [53]. In line with this, T-006 upregulated mTOR and p62 as well as Nrf2/HO-1 levels, providing further evidence for the effectiveness of T-006 as a prospective antioxidative treatment for neuroprotection.

In addition, we investigated the protective effects of T-006 by using mouse brain endothelial cells bEnd.3 challenged with OGD/R. This model has been widely accepted for studying BBB disruption after ischemia. The occurrence of ischemic stroke is associated with BBB disruption and tight junctions (TJs) breakdown [3]. bEnd.3 exposed to OGD/R exhibited a significant increase in permeability, decreased TJs protein levels, reduced cell viability, and the upregulation of pro-apoptotic protein expression. These changes are critical indicators of endothelial dysfunction and BBB disruption, both of which are central to the pathology of ischemic stroke. These alterations in bEnd.3 mimic the physiological responses seen in vivo during ischemic events and serve as a valuable platform for investigating antioxidant therapeutics in preserving endothelial functions, integrity, and cell survival. More importantly, previous studies have demonstrated that protecting endothelial function could effectively ameliorate the damage caused by ischemic stroke [15]. Our result showed that T-006 alleviated OGD/R-induced bEnd.3 cells injuries. Results from Western blotting demonstrated that T-006 enhanced the expressions of ZO-1 (TJ protein) and Nrf2 and HO-1 (antioxidative stress proteins) and p-AKT, which was inhibited by the PI3K inhibitor LY292004. These data suggested that T-006 could protect endothelial cell function as well as TJ integrity.

Ischemia is a complex pathological process that involves damage to both neuronal and endothelial cells [1,54,55]. In neuronal cells, T-006 protects from oxidative injury by modulating the autophagy and antioxidant pathways. In endothelial cells, T-006 primarily enhances antioxidant pathways to combat oxidative damage. Given that endothelial cells are particularly susceptible to oxidative stress during ischemic stroke, these antioxidant mechanisms are crucial for maintaining the integrity of the blood-brain barrier. In contrast, oxidative injury in neurons often results from secondary effects, such as increased glutamate levels and calcium dysregulation, necessitating a robust cellular defense against apoptotic cell death due to mitochondrial damage. Thus, regulating autophagy is vital for neuronal protection. Thus, we propose that T-006 is highly beneficial in ischemic stroke, as it addresses the specific vulnerabilities of both cell types through multifaceted molecular mechanisms.

## 5. Conclusions and Future Perspectives

Taken together, our present study demonstrated that T-006 effectively protected both neuronal and brain endothelial cells under oxidative stress challenge in ischemic hypoxia. Further studies using relevant human neuronal and brain endothelial cell models, such as induced pluripotent stem cells (iPSCs), from patients with neurodegenerative diseases and ischemic stroke, will improve the understanding of the therapeutic potential of T-006. Additionally, incorporating multi-omics analyses and single-cell RNA sequencing will shed light on the molecular mechanisms of the compound and the interactions between neurovascular cells in the brain.

## Data Availability

Data are contained within this article.
